# Sorghum’s Whole-Plant Transcriptome and Proteome Responses to Drought Stress: A Review

**DOI:** 10.3390/life11070704

**Published:** 2021-07-17

**Authors:** Rudo Ngara, Tatenda Goche, Dirk Z. H. Swanevelder, Stephen Chivasa

**Affiliations:** 1 Department of Plant Sciences, University of the Free State, Qwaqwa Campus, Phuthadithjaba 9866, South Africa; 2 Department of Crop Sciences, Gwanda State University, Epoch Mine Campus, Filabusi, Zimbabwe; tatendagoche@gmail.com; 3 Agricultural Research Council’s Biotechnology Platform, ARC Onderstepoort Campus, Onderstepoort 0110, South Africa; SwanevelderD@arc.agric.za; 4 Department of Biosciences, Durham University, South Road, Durham DH1 3LE, UK; stephen.chivasa@durham.ac.uk

**Keywords:** sorghum, drought stress, whole-plant responses, systems biology, transcriptomics, proteomics, differentially expressed genes, differentially accumulated proteins

## Abstract

Sorghum is a cereal crop with key agronomic traits of drought and heat stress tolerance, making it an ideal food and industrial commodity for hotter and more arid climates. These stress tolerances also present a useful scientific resource for studying the molecular basis for environmental resilience. Here we provide an extensive review of current transcriptome and proteome works conducted with laboratory, greenhouse, or field-grown sorghum plants exposed to drought, osmotic stress, or treated with the drought stress-regulatory phytohormone, abscisic acid. Large datasets from these studies reveal changes in gene/protein expression across diverse signaling and metabolic pathways. Together, the emerging patterns from these datasets reveal that the overall functional classes of stress-responsive genes/proteins within sorghum are similar to those observed in equivalent studies of other drought-sensitive model species. This highlights a monumental challenge of distinguishing key regulatory genes/proteins, with a primary role in sorghum adaptation to drought, from genes/proteins that change in expression because of stress. Finally, we discuss possible options for taking the research forward. Successful exploitation of sorghum research for implementation in other crops may be critical in establishing climate-resilient agriculture for future food security.

## 1. Introduction

Sorghum (*Sorghum bicolor*) is the world’s 5th most important cereal in terms of area harvested and yield [1]. Together with pearl millet (*Pennisetum glaucum*) [2], sorghum grows well in hot and dry environments less suitable for other major cereals, such as maize (*Zea mays*) and rice (*Oryza sativa*) [3,4]. The sorghum plant has many uses, with its grain used as a source of food and feed, while stalks provide raw materials for building, broom-making, and biofuel production [3,4,5]. Sorghum germplasm is genetically diverse, with large active and base collections of landraces, improved breeding lines/cultivars, and wild relatives being maintained across several genebanks worldwide [6,7,8]. This rich gene pool provides essential genetic variation required for sorghum research, breeding, and crop improvement programs [8,9].

Despite sorghum’s economic importance [5] and rich genetic diversity [3,6,7,8], its genetic potential is relatively underused compared to other major cereals [2]. Sorghum production is also constrained by several biotic and abiotic stresses [3,4,8,10]. Among the latter, drought and heat stresses can significantly reduce the yield of some sorghum varieties, especially in rain-fed agricultural systems [11,12]. Heat and drought spells are also predicted to increase in occurrence with global warming [13,14], further constraining crop productivity of subsistence farmers in drought-prone areas. In such communities, adequate food provision will become even more challenging due to a combination of socio-economic factors, unpredictable climates, and the use of inferior crop varieties [12]. Additionally, with the ever-increasing population, food insecurity is now a global concern, and agricultural scientists are looking at underused but climate-resilient crops and varieties, including those of sorghum, for cultivation in marginal lands [2].

Sorghum is generally regarded as being drought-resilient; however, the degree of tolerance varies between species, genotypes/accessions, the prevailing environmental conditions, and their complex interactions [3,4]. Plants are therefore still vulnerable to drought stress at some point during growth [10,15], with exposure and different susceptibilities to moisture stress at these developmental stages potentially disrupting seed germination, plant establishment, growth, flowering, and/or grain filling processes, ultimately reducing grain size and yield [10,15,16,17]. In extreme cases, plant death and complete crop failure may occur [10,17]. However, the varying degrees of accession tolerances result in some capable of withstanding soil moisture stress during the pre-flowering stage, while others provide tolerance in the post-flowering growth stages [16,18,19]. Pre-flowering drought-tolerant lines maintain normal panicle development when exposed to water deficits during the pre-flowering phase [16]. In contrast, post-flowering drought-tolerant lines are associated with the stay-green trait, retaining high chlorophyll content and photosynthetic activity after anthesis during terminal drought [16,20,21,22]. Such genetic diversity is invaluable for environment-specific crop breeding programs.

**Figure 1:**
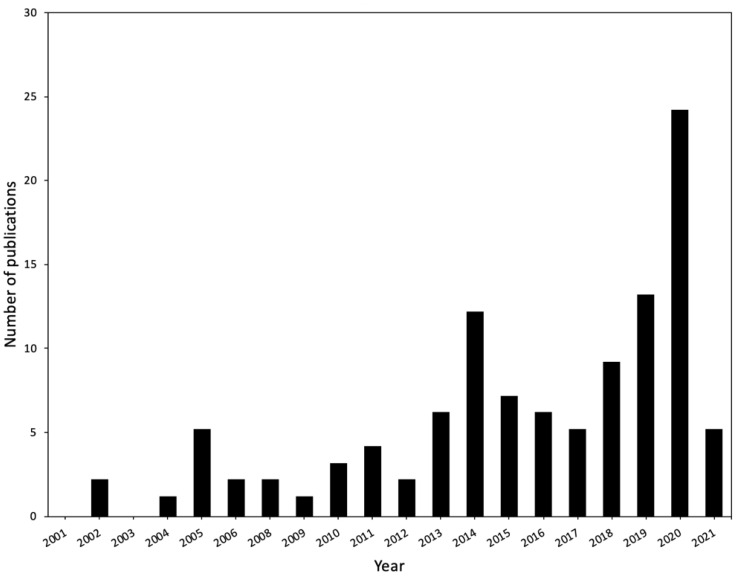
Number of publications per year related to sorghum drought stress since 2001. Keywords used in searches on the PubMed database included “*sorghum water limitation*, *sorghum water deficit*” with additional terms, such as “*transcriptomics, proteomics, metabolomics*”. Database was accessed on 14 March 2021.

Apart from this wide gene pool, the sorghum genome has been sequenced [23] and is available on online databases such as Phytozome [24] and the National Center for Biotechnology Information (NCBI) [25] (Table 1). In general, the availability of crop genome sequences is a major milestone in agricultural biotechnology, which enables the identification of genes associated with important agronomic traits [26,27,28]. Its small genome size (±730 Mb) make sorghum an ideal model plant for comparative functional genomics in grasses [23,29,30], for understanding the C_4_ photosynthesis pathway, and the drought tolerance trait [23,26]. To aid the scientific community in this endeavor, some computational tools have been developed to support “omics” (genomics, transcriptomics, proteomics, metabolomics and bioinformatics) research of this crop (Table 1). Consequently, just over a decade after the release of the first draft sorghum genome sequence [23], research interest in “omics” studies of sorghum in response to abiotic stresses is steadily increasing (Figure 1). Therefore, we review drought transcriptomics and proteomics studies of sorghum whole-plant systems and highlight target genes and proteins for further functional studies prior to their application in crop breeding programs.

## 2. General Plant Responses to Drought Stress

Water is essential to all life forms. It maintains cell turgidity and structure in plants and is a basic requirement for growth, physiological and biochemical processes [55]. Nonetheless, different plant species may have varying water requirements to support normal growth and development. For example, among cereal crops, sorghum requires less water than maize, barley (*Hordeum vulgare*), and wheat (*Triticum aestivum*) [3]. However, as stated earlier, most plant species experience some level of drought stress (also known as water stress, osmotic stress, dehydration stress, or water deficits) at one or more growth stages during their life cycles. The term ‘drought’ describes periods of insufficient soil moisture content to meet the needs of a plant [56]. Consequently, plants alter their morphology, physiology, and biochemistry to mitigate the damage caused by primary and secondary effects of water scarcity and resume growth [57,58,59,60]. These plant responses are broadly categorized as drought escape, avoidance, and tolerance mechanisms and have been extensively described in [57,61,62,63]. Drought escape refers to plants remaining dormant during periods of water stress or rapidly completing growth cycles before the onset of the drought season [57,61,62]. Drought avoidance involves morphological/anatomical changes to allow the maintenance of high plant water potential during drought [57,61] and is primarily achieved in one of three ways: Maintaining high water uptake through an extensive root system; Storing water in succulent tissues, and reducing transpiration water loss through numerous leaf modifications and adaptations that decrease total leaf surface area, and/or increase stomatal control and cuticular resistance [57,61,62]. However, drought tolerance mechanisms involve stress recognition and signaling events, followed by alterations in gene, protein and metabolite expression to alleviate the stress damage and regulate stress-inducible gene expression [58,59,64,65].

Water deficits also affect the biosynthesis and catabolism of several hormones in plant tissues which in turn modulate many physiological processes [62]. For example, during drought stress, abscisic acid (ABA) and ethylene levels increase, while those of gibberellins, auxins and cytokinins decrease [55,61,62]. ABA, a well-known root-to-shoot stress signaling compound controls stomatal closure and the expression of stress genes during drought [66], while ethylene functions in leaf abscission [61]. These processes are important in conserving water and alleviating osmotic stress damage imposed by drought.

Likewise, sorghum responses to drought stress involve changes in morphology, physiology, and/or molecular activities. Amelework et al. [67] published a comprehensive review of physiological mechanisms employed by sorghum plants in response to water deficits and is highly recommended for further reading. Another review focused on general responses of sorghum to abiotic stresses [68], while Blum [69] compiled a comprehensive book chapter on sorghum physiology under normal growth conditions and in response to stress factors. Some drought avoidance mechanisms of this crop include changes in root structure, distribution, and depth [70], a decrease in stomatal conductance [71], retention of chlorophyll content [72], increased biosynthesis of epicuticular wax [73,74], and its deposition on leaf blades [75,76].

Undoubtedly, whole-plant responses to drought stress are complex and involve the coordinated responses of tissues, cells, their compartments and composite macromolecules [77]. Consequently, plant scientists are now employing systems biology approaches [78], including “omics” technologies [79], to study the spatial and temporal expression changes of transcripts, proteins, and metabolites in response to abiotic stresses [80]. Such experimental approaches are both integrative and complementary, as they seek to unravel the network of biochemical pathways, their interactions, dynamics, and regulatory nodes during stress response [78]. Cramer and co-workers [80] have already given an excellent review of systems biology studies of plants in response to abiotic stress, and is recommended for further reading. However, similar reviews on sorghum are limited. Therefore, in the current review, we focus on studies that have employed transcriptomics and proteomics workflows to understand how sorghum plants respond to limited water supply.

## 3. Sorghum Transcriptomics Studies in Response to Drought Stress

Transcriptomics is the large-scale analysis of expressed RNAs in an organism, at a particular moment in time of a growth stage and/or in response to an environmental stimulus [81,82]. Although mRNAs are translated into proteins, non-coding sequences play critical regulatory functions during normal plant growth, including stress responses [83,84]. Techniques used to study transcripts are diverse, ranging from Northern blotting and hybridization methods, expressed sequence tags (ESTs), reverse transcriptase quantitative PCR (RT-qPCR), serial analysis of gene expression (SAGE) to RNA microarray, and RNA sequencing (RNA-seq) (reviewed by Lowe et al. [82]). Currently, microarrays and RNA-seq techniques are most commonly used in studies of the plant transcriptome, and each method has its pros and cons [82].

Transcriptomic studies of sorghum plants under drought stress have also used a broad spectrum of RNA profiling techniques, plant tissues, experimental designs and water stress treatments [19,73,74,85,86,87,88,89,90,91,92,93,94], as summarized in Table 2. Earlier studies investigated a dehydrin mRNA expression pattern in drought-stressed sorghum tissues using Northern blotting and hybridization methods against a ^32^P labeled maize dehydrin cDNA probe [85,86]. Dehydrins are late embryogenesis-abundant (LEA) D-11 family proteins that accumulate in dehydrated tissues and possibly act as stabilizers of cell components [95,96]. Cheng and co-workers [85], exposed 10-day old sorghum seedlings to a slow dehydration process in a closed chamber with 3M NaCl, and sampled shoots over 22 h. Results showed the presence of a drought-induced sorghum dehydrin transcript, whose accumulation increased with prolonged exposure to the dehydration stress. Similarly, Wood and Goldsborough [86] reported increased levels of a dehydrin (*DHN1*) mRNA in both sorghum seedlings and mature plants exposed to water limitation. However, this transcript was not detected in well-watered plants or those recovering from drought. The authors concluded that both the *DHN1* mRNA and its protein are drought-inducible in sorghum tissues [86] and possibly hold a central role in drought adaptation [95,96].

Apart from anchoring plants into the soil, roots also play a critical role in drought stress signaling processes. They sense the development of soil moisture deficits and transmit signals—including ABA, to other plant tissues to elicit stress responses [66,97]. Buchanan et al. [87] conducted a cDNA microarray-based transcriptomics study of shoots and roots of the sorghum cultivar BTx623 in response to 20% polyethylene glycol (PEG)-induced osmotic stress, NaCl-induced salinity, and exogenous ABA. Tissue sampling was done at 3 and 27 h to investigate temporal changes in transcript expression and to reduce circadian cycling interference on the data. The study reported about 2200 differentially expressed genes (DEGs) in response to the three different treatments, with some being unique to a plant tissue, treatment, or sampling time point. Collectively, the DEGs had putative functions in signaling, regulation and gene expression, growth, metabolism, transport, protection from dehydration, reactive oxygen species (ROS) detoxification, and plant defense, while other genes had unknown functions. Examples of up-regulated genes include those of various transcription factors, signaling proteins, LEAs, dehydrins, heat shock proteins (HSPs), small HSPs, expansins, and several ROS detoxification enzymes. In addition, the expression levels of genes involved in the biosynthesis of ABA, proline, and the raffinose family of oligosaccharides (RFOs) were also enhanced [87].

However, since the microarray-based study by Buchanan and co-workers [87] was conducted prior to the release of the sorghum genome sequence [23], it was later discovered that the sorghum-microarray probes used had limited gene coverage [88]. Expanding on their previous study, Dugas and co-workers [88] conducted RNA-seq analyses of roots and shoots of the same sorghum variety in response to 20% PEG, and exogenous ABA treatments. Samples were collected after 27 h to mimic the research group’s earlier study [87]. The study found 28,335 genes with transcriptional activity, of which between 1000–3200 genes were differentially expressed depending on the tissue type or treatment. The results also revealed that exogenous ABA modulated the expression of a greater number of genes than PEG. In addition, between 12–30% of the DEGs were common to both treatments depending on the tissue type, possibly indicating the central role of ABA in osmotic stress response. Examples of the DEGs included those involved in the biosynthesis of several plant hormones, metabolism of amino acids and other osmoprotectants, cell growth, ROS detoxification, and defense pathways against pathogen attack. The families of up-regulated transcripts were generally similar to those previously reported by Buchanan et al. [87] including a wider pool of transcription factors, a signaling CLAVATA3 protein, the water stress-inducible protein 18 (WSI18) and biosynthesis enzymes of the osmoprotectant β-alanine betaine [88].

Similarly, Zhang and co-workers [90] investigated the transcriptomic profiling of leaves and roots of a drought-tolerant sorghum variety XGL-1 in response to drought stress using RNA-seq analysis. Sorghum seedlings were exposed to either mild or severe water deprivation for 7 days before re-watering some for an additional 2 days. Control plants were well-watered throughout the experiment. Between 19,000–22,000 genes were identified in the two tissue types, of which 13,285 were differentially expressed in response to the various treatments. In general, roots exhibited more DEGs than leaves, possibly implicating roots in stress perception and relay of signals during drought response. Gene Ontology (GO) enrichment analyses revealed overlaps in the over-represented biological processes between roots and leaves, including response to stimuli, such as ABA, temperature, and light intensity, response to water deprivation, and response to oxidative stress. In addition, root samples had DEGs with carbohydrate-related metabolic processes such as sucrose metabolic process and raffinose family oligosaccharide biosynthetic process which were not detected in leaves. Such tissue-specific gene expression patterns possibly highlight the increased importance of carbohydrates in roots during drought response and/or the putative functions of sugars in signaling processes.

Other remarkable observations in the study [90] relate to a shift in hormonal balance during drought and recovery from stress. For example, DEGs involved in ABA biosynthesis and catabolism were up-regulated in roots during drought, but down-regulated during recovery from stress, with an inverse expression pattern of DEGs involved in the auxin signal pathway. These results underscore the distinctive roles of various plant hormones in response to water deficits [55,62]. In addition, it is well-known that plants exposed to environmental stresses tightly regulate gene expression to re-direct plant resources for the maintenance of critical cell processes and homeostasis [65]. Zhang et al. [90] also identified DEGs of transcription factors (TFs) in response to water deficits, with only four in leaves but 66 in roots. This observation further highlights the central role of the root transcriptional activities in driving plant responses to water deficits. The identified TFs possibly regulate the expression of many functional and protective genes and downstream proteins required for drought response and adaptation.

Leaves are the prime photosynthetic tissue of most crops and also serve as sites for gas exchange and transpiration [55]. Drought stress affects carbon assimilation and fixation [62], which contributes to reduced plant growth and yield [11]. Furthermore, heat and drought stresses are usually concurrent under field conditions, exerting even greater detrimental effects on plant growth than each stress alone [98,99]. Both stresses cause the over-production and accumulation of reactive oxygen species (ROS) in cells [100], which lead to oxidative damage of macromolecules and photosynthetic machinery [101,102]. As with other stress factors, plants respond to the effects of drought and/or heat stresses by reprogramming gene expression [100]. Consequently, Johnson and co-workers [73] investigated the transcriptional responses of leaves of R16 sorghum seedlings in response to drought, heat, and a combination of the two stresses using DNA-microarray analysis. The microarray chip consisted of 28,585 gene spots, which correlated with the draft sorghum genome at the time. In the study, 14-day old seedlings were either exposed to drought by withholding water for 3 days at 28 °C, 3 h heat shock treatment at 50 °C, and a combination of the two stresses while well-watered controls were kept at 28 °C. The study reported transcriptional changes in about 3.5%, 18% and 22% of the genes on the microarray chip in response to drought, heat and combined stresses, respectively. Examples of the highly up-regulated transcripts in response to drought included genes of LEA proteins, *P5CS2*—a proline biosynthetic gene, and *HKT1*—a sodium ion transmembrane transporter. GO enrichment analyses also identified the over-representation of genes involved in responses to stress such as water deprivation, responses to ABA, regulation of photosynthesis, fluid transport, and amino acid metabolism. In addition, promoter motif analysis of the up-regulated drought-responsive genes identified the ABA-responsive element (ABRE) motif as highly represented.

Although overlaps were observed in some DEGs across the three treatment groups (drought, heat, and a combination of the two), others were unique to each of the treatments [73], signifying crosstalk and specificity in plant responses to an array of stresses [103]. For example, 380 genes were up-regulated only in the drought-stressed plants, including transcripts of specific LEA proteins, expansins, lipid transport, and lipid transfer proteins. Interestingly, when Johnson et al. [73] compared their sorghum drought-responsive transcripts to those reported by Dugas et al. [88] in response to 20% PEG-induced osmotic stress, only a third of the genes where common between the two studies. This indicates that different types of water deficit treatments may trigger different response pathways [73]. As reviewed by Osmolovskaya and co-workers [104], drought models used in plant studies are diverse, with each set-up potentially exerting intrinsic effects of water limitations to the experimental plant system—leading to somewhat heterogeneous results. Consequently, the most enriched biological processes in the study by Dugas and co-workers [88] included responsive to stress, and ROS, while wax biosynthetic processes were over-represented in the study by Marc Knight’s group [73].

Landraces are rich sources of untapped genetic variation that can be harnessed for the development of elite crop cultivars [6]. Accordingly, Denvarain et al. [89] analyzed changes in the leaf transcriptome of a South African sorghum landrace LR6 in response to water deficits using cDNA microarrays. The plants were exposed to three watering regimes—mild stress, severe stress and re-watering, and all transcripts were compared to those of well-watered controls. Out of the 35,899 transcript probes on the sorghum-microarray chip, a total of 368, 414, and 137 were differentially expressed in response to the mild stress, severe stress, and re-watering conditions, respectively. Although the DEGs covered a wide range of functional categories, TFs largely dominated the drought-responsive transcripts identified in the study and were more prominent in severely stressed leaves. These results underscore the critical role of TFs in regulating gene expression during drought response. Similar to other transcriptomics studies reviewed above, the significantly enriched transcripts belonged to the following biological processes: response to abiotic stimulus, homeostatic process, regulation of biological quality, cellular homeostasis, and response to stimulus. Similar to reports by Dugas et al. [88] genes involved in the biosynthesis of β-alanine betaine were also up-regulated [89].

Although the studies reviewed above provided invaluable information on DEGs in drought response of plant tissue(s) from a single sorghum variety, comparative studies between two [19,74,92,93] or more [91,94] genotypes with contrasting drought phenotypes are important in identifying drought tolerance-related genes for potential application in molecular breeding [62]. On this premise, Fracasso and co-workers [74] used RNA-seq to study the global transcriptome changes of leaf meristems in two sorghum varieties with contrasting water use efficiency in response to drought. Both genotypes were exposed to similar levels of water deprivation before RNA sequencing, and data analyses were extensively performed within and between genotypes to identify trends in constitutive gene expression versus those induced by drought. The results revealed that water deficits significantly increased alternative splicing events in the drought-tolerant genotype (IS22330) compared to the sensitive genotype (IS20351). Alternative splicing is one of many gene regulatory mechanisms employed by plants to increase protein diversity and function during normal growth/physiological processes, and in response to adverse environmental conditions [105,106,107], and may contribute towards drought resistance of some sorghum genotypes.

Some overlap in DEGs and GO terms were also identified between the two genotypes. However, the authors highlighted a particularly high constitutive expression of genes involved in secondary metabolism, including those of non-enzymatic antioxidant compounds and glutathione transferase (GST) activity in the tolerant genotype, possibly contributing to its inherent drought-superior traits compared to the sensitive genotype [74]. The authors argue that for this reason, the drought-tolerant genotype exhibited a lesser number of DEGs in response to drought, totaling 636, compared to 1599 in the drought-sensitive genotype. Furthermore, GO terms such as response to abiotic stimulus, oxido-reductase activity and response to stress were significantly down-regulated in the susceptible variety compared to the tolerant type. However, cuticular wax biosynthesis genes remained unchanged in the tolerant genotype in response to drought stress but were elevated in the drought-sensitive genotype, possibly to increase wax deposits for improved water conservation [75].

Likewise, Azzouz-Olden et al. [92] conducted a comparative RNA-seq analysis of sorghum leaf tissue following post-anthesis drought in two sorghum genotypes with contrasting tolerance to post-flowering drought stress. Similar to the results of Fracasso and co-workers [74], the authors [92] reported higher constitutive expression of genes involved in redox homeostasis, translation, and the biosynthesis of a range of metabolites in the drought-tolerant genotype SC56 compared to Tx-7000, which is sensitive to post-flowering drought stress. Following water deprivation, the drought-tolerant genotype exhibited the up-regulation of antioxidation-related genes such as GST, superoxide dismutase, peroxidases, biosynthetic enzymes of non-enzymatic antioxidants (such as tocopherols and glutathione), and genes involved in transmembrane transporters. Thus, biological processes that are either highly constitutively expressed or over-represented in the drought-tolerant genotypes during water stress compared to the sensitive genotypes could possibly represent putative genes that confer drought tolerance traits in these sorghum genotypes.

Some sorghum genotypes tolerate either pre-flowering or post-flowering drought stress and may exhibit differential responses to water deficits during these developmental stages [16]. Varoquaux et al. [19] conducted a large-scale transcriptome analysis of two field-grown sorghum genotypes in response to pre- and post-flowering drought stress, as well as recovery from pre-flowering drought. The genotypes used, RTx430 and the stay-green BTx642, are tolerant to pre-flowering and pre-flowering drought stress, respectively. Leaf and root samples of both genotypes were collected at weekly intervals over 17 weeks and analyzed for drought transcriptome changes relative to those of well-watered controls. RNA sequencing was performed on 198 root and 198 leaf samples, followed by GO and enrichment analyses. The study revealed a large transcriptional response to drought, with 10,727 genes accounting for 44% of the expressed genes showing changes in response to the various treatments. However, 10% of the expressed genes showed changes to water deprivation within the first week of the drought treatment, indicating a quick response to water deficits.

Furthermore, after re-watering, about 75% of the pre-flowering drought-responsive genes had similar expression levels to those of well-watered control plants. As reported by Zhang and co-workers [90], root tissues exhibited a greater number of DEGs than leaves, possibly reinforcing the role of roots in drought sensing and signal transduction pathways [19]. The study’s other key findings include differences in constitutive and drought-induced transcriptional activities between the two genotypes, including GST and proline biosynthetic genes. Overall, this comprehensive transcriptome study revealed the complex spatial and temporal drought responses between genotypes with contrasting phenotypes.

In another comparative study, Abdel-Ghany and co-workers [91] exploited the vast genetic diversity of sorghum and analyzed transcript changes of drought-stressed 8-day old seedlings of four genotypes. Osmotic stress was imposed using 20% PEG, and samples were assessed for early (1 h) and late (6 h) changes in the transcriptome using RNA-seq. The four genotypes used, Bx623, SC56, Tx-7000, and PI482662, were previously reported to possess some level of drought tolerance either at pre-anthesis and pre-flowering or post-flowering growth stages (see references in [91]). However, the authors categorized the genotypes as drought-resistant (Bx623 and SC56) and sensitive (Tx-7000 and PI482662) based on root length traits in response to PEG-induced osmotic stress [91]. Five notable trends emerged from this study. All four sorghum genotypes responded to PEG-induced osmotic stress by up-regulating a greater number of DEGs at 6 h. Unique and common DEGs were reported across all genotypes, and some common DEGs are well-known drought-responsive genes such as those of TFs, hormone signaling, stress, and detoxification/antioxidant processes. Early responses to PEG-induced osmotic stress revealed the involvement of genes associated with ABA, jasmonic acid, and auxin hormone signaling and TFs, while late responses included genes involved in abiotic stress, secondary metabolism, heat shock, and GST synthesis. However, limited overlap between DEGs at the two, time points was observed. Some of the DEGs unique to the drought-resistant lines Bx623 and SC56 included LEA genes, TFs, signaling, and lipid metabolism—and these could be involved in conferring drought tolerance to the drought-resistant sorghum lines.

The non-coding RNAs are increasingly being studied for their role in post-transcriptional regulation. Katiyar et al. [93] investigated the regulatory roles of two small, non-coding RNAs (sRNAs), namely microRNAs (miRNAs) and trans-acting small interfering RNAs (tasi-RNAs), in sorghum drought-responsive gene expression. The study compared sRNA libraries from susceptible (C43) and drought-tolerant (M35-1) sorghum lines under both drought stress and control conditions. More than 500 novel miRNAs were identified, but only 96 were drought-unique, with 32 up-, 49 down-regulated (≥2-fold change), and 15 genotype-contrasting drought-regulated expression patterns. Of these 96 miRNAs, 63 showed genotype opposing regulation. Of these, 44 genes were up-regulated in the drought-tolerant genotype, but down-regulated in the susceptible (C43) genotype, while 19 were down-regulated M35-1 and up-regulated in C43. Genotype-dependent drought stress responses were also observed, with 17 miRNAs differentially regulated in the sensitive genotype and 18 miRNAs in the tolerant genotype. A total of 1300 potential genomic targets were predicted for the novel and known 432 miRNA families identified, with no targets identified for the remaining 96 new miRNA families. Predicted miRNAs were involved in the regulation of metabolic, cellular, and biological processes, and responses to stimuli, with a number identified previously in both sorghum and other crops to be active during drought stress. Two *TAS3* gene orthologs, targeted by miR390, were identified and together confirmed the presence of a sorghum miR390-Tas3 pathway and a possible role in the auxin signaling pathway. Six miRNA families were predicted to target peroxidases, i.e., enzymes involved in restricting ROS build-up.

Hamza et al. [94] profiled 11 elite sorghum lines with 8 miRNAs previously shown to be down-regulated during abiotic stress. The selected miRNAs had predicted target transcripts across 66 different GO terms, with the main categories belonging to biological processes, cellular components, and molecular function. The 8 selected miRNAs’ expression profiles were compared under well-watered control and drought stress conditions. The results showed that miRNA profiles varied largely between genotypes, and no uniform miRNA expression pattern could be identified. Based on this, the authors suggested that sorghum drought tolerance could be a “fine-tuning mechanism” that varies between genotypes, therefore influencing growth and developmental processes—especially since many of the miRNAs studied had links to auxin signaling and ROS [94].

## 4. Sorghum Proteomics Studies in Response to Drought Stress

Proteomics is the large-scale analysis of expressed proteins in an organism, tissue, cell, or cellular compartment during normal development or in response to the changing environment [108,109]. As reviewed previously [110,111], proteomics technologies are diverse and broadly categorized into gel-based and non-gel-based techniques, coupled with mass spectrometry for protein identification and downstream bioinformatics analyses for putative functional characterization of the identified proteins. Earlier reviews have discussed the progress made in plant proteomics under a range of abiotic stresses and can be accessed for further reading [111,112,113,114]. In this section, we summarize proteomic studies of sorghum plants under drought stress. Compared to the sorghum whole-plant transcriptomic analyses discussed above, the complementary proteomic work of this crop is extremely limited to a few publications [71,115,116,117] (Table 3). This highlights the need for more studies to analyze the dynamics of sorghum proteomes in response to water deprivation as we seek to understand the molecular networks that contribute to the crop’s natural resilience to drought.

In a pioneering proteomic study of sorghum leaves under drought stress, Jedmowski et al. [115] conducted a comparative two-dimensional difference gel electrophoresis (2D DIGE) analysis between drought-sensitive, 11,431 and tolerant, 11,434 sorghum accessions in drought-stressed and re-watered plants. The study reported proteome changes in both accessions in response to the treatments, with common and unique drought-responsive protein and/or expression patterns. The identified proteins have putative roles in metabolism, energy, transcription, protein synthesis, protein destination and storage, while some were unclassified. Further analysis of the drought-responsive proteins revealed that the drought-sensitive variety has increased levels of proteolytic enzymes, while those involved in transcriptional activity, synthesis, and stability of proteins were enhanced in the drought-tolerant sorghum variety.

Goche et al. [71] conducted a comparative root proteome analysis of two sorghum varieties in response to drought stress using the gel-free isobaric tags for relative and absolute quantitation (iTRAQ) technology. The sorghum varieties used ICSB338, and SA1441 are susceptible and tolerant to drought, respectively. In the study, drought stress was simulated by withholding water to young seedlings for 8 days prior to analyzing the root proteome. Of the 1169 and 1043 positively identified root proteins in the drought-sensitive and drought-tolerant varieties, 237 and 187 were responsive to drought, respectively. Further comparative analysis of these stress-responsive proteins revealed that 51 were common to both varieties, albeit with some differences in abundance levels. The rest, 186 and 136, were unique to ICSB338 and SA1441 sorghum varieties, respectively. Although the majority (>70%) of the positively identified proteins were uncharacterized, bioinformatics tools were used to group the drought-responsive proteins into theoretical functional categories such as defense/detoxification, proteolysis, transporter/intracellular transport, metabolism, transcription, protein synthesis, proteolysis, signal transduction, and cell structure.

Further interrogation of the drought-responsive proteins revealed several notable trends within and between the two sorghum varieties [71]. For example, among the common proteins, those involved in signal transduction and defense/detoxification-related processes were generally up-regulated, but metabolism-related proteins were mostly down-regulated. The unique proteins also depicted contrasting expression dynamics, which possibly contribute towards the differential performance of these two varieties under water-limited conditions. For example, the drought-superior variety, SA1441 responded to water deficits by increasing the abundance of proteins related to transcription, protein synthesis, protease inhibition, signaling, defense/detoxification-related processes compared to the drought-susceptible ICSB338. Conversely, ICSB338 increased the accumulation of proteolytic enzymes while down-regulating those involved in metabolism and protein synthesis. Thus, both proteomic studies [71,115] highlight the importance of drought-induced transcriptional activity, and protein synthesis in increasing the pool of proteins, and downstream metabolites with protective and signaling roles during drought stress. In contrast, increased protein degradation and reduced metabolism are common responses among the drought-sensitive genotypes. However, further functional studies are required to elucidate the roles of these proteins in drought response.

Another recent study [116] also analyzed the root proteome changes of the Bx623 sorghum inbred line in response to a 24 h treatment in 10% PEG-induced osmotic stress using 2D gel electrophoresis and matrix-assisted laser desorption/ionization time-of-flight tandem mass spectrometry (MALDI-TOF-TOF MS) analysis. The study reported 105 differentially accumulated proteins in response to the PEG treatment, with 65 of these with a 2-fold change in abundance. Of these proteins, 43 were up-regulated and 22 down-regulated. The majority of the stress-responsive proteins were involved in energy and carbohydrate metabolism, antioxidant and defense response, and protein synthesis/processing/degradation, further complementing a previous root proteomic study [71]. As observed in the comparative transcriptomics studies, putative functional groups of stress-responsive proteins identified in the drought-tolerant varieties possibly contribute to the tolerant nature of the sorghum accessions by producing proteins/metabolites responsible for maintaining cellular homeostasis under water stress.

## 5. An Overview of Sorghum Molecular Responses to Drought Stress

**Figure 2:**
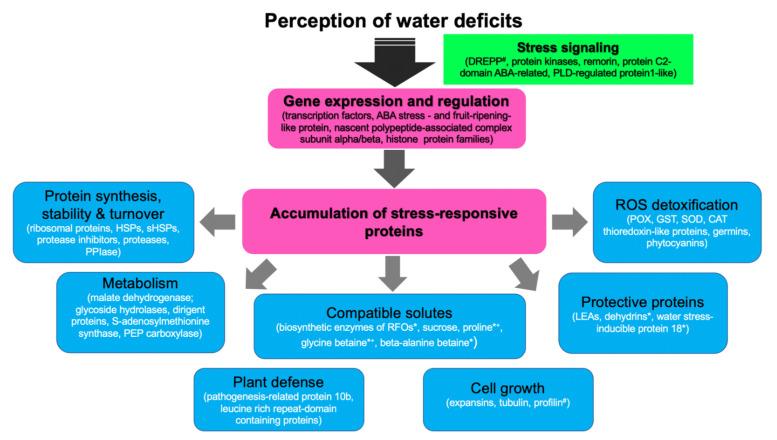
A generalized molecular response network of drought-stressed sorghum plants. Up-regulated root and leaf proteins of drought-tolerant sorghum genotypes were obtained from two drought proteomics studies [71,115] and their protein family names used to search similarly up-regulated genes in transcriptomics datasets [73,88]. Common gene families between the transcriptomics and proteomics studies were used to construct the diagram. ^#^ denotes proteins identified in the proteomics studies, * denotes genes identified in transcriptomics studies, *^+^ denotes biosynthesis enzymes of compatible solutes identified in the transcriptomics studies, while the solute levels were reported by Goche et al. [71]. ABA, abscisic acid; CAT, catalase, DREPP, developmentally regulated plasma membrane polypeptide; GST, glutathione transferase; HSPs, heat shock proteins; sHSPs, small heat shock proteins; PEP, phosphoenolpyruvate; PLD, phospholipase D; POX, peroxidase, PPIase, peptidyl-proline isomerase, RFOs, raffinose family of oligosaccharides; ROS, reactive oxygen species; SOD; superoxide dismutase.

In summary, sorghum molecular responses to water deprivation involve stress perception/signaling events followed by gene, protein, and metabolite expression changes. The reviewed transcriptomics [19,73,74,85,86,87,88,89,90,91,92,93,94] and proteomics [71,115,116,117] studies also affirm such complexities in drought tolerance mechanisms of the crop. However, because the studies employed a wide range of experimental designs, detailed comparisons between individual drought-responsive genes and proteins are difficult to perform across experiments. Nonetheless, we illustrate a generalized molecular response network of drought-stressed sorghum plants (Figure 2) using published [71,115] up-regulated proteomes of drought-tolerant sorghum varieties. These proteomic data are also largely supported by drought-responsive sorghum transcriptomes reported by Dugas et al. [88] and Johnson et al. [73].

As expected, transcriptomics studies reported a larger pool of drought-responsive transcription factors than proteomics studies, which drive gene expression changes. Some of the identified stress-responsive proteins are possibly involved in protein synthesis, stability and turnover events, primary and secondary metabolism, cell growth processes, osmoregulation, protective functions against dehydration, plant defense, and ROS detoxification processes (Figure 2). Although this illustration is not exhaustive, it shows well-known drought-responsive genes/proteins previously identified in Arabidopsis and other plants, including cereals [60,64,65,100]. Furthermore, the identification of β-alanine betaine biosynthesis enzymes in the sorghum drought-responsive transcriptome [88,89] also provides new insights into the possible functions of this solute in the crop. Beta-alanine betaine in known to accumulate in the stress-tolerant plant family Plumbaginaceae where it possibly serves as an alternative osmoprotectant to glycine betaine under salinity and hypoxic conditions [118,119,120,121]. However, more functional studies are required to validate these drought-responsive genes/proteins in sorghum.

## 6. Conclusions and Future Perspectives

Plants, including sorghum respond to water deprivation through complex alterations in gene and protein expression, with concomitant changes in whole-plant physiology and metabolism. Comparative “omics” studies, using sorghum varieties with contrasting drought phenotypes, have broadened our knowledge of cellular processes that potentially contribute towards the inherent superior nature of some sorghum genotypes. For example, under well-watered conditions, drought-tolerant sorghum exhibits higher constitutive expression of genes associated with secondary metabolism and ROS detoxification-related pathways. In addition, upon exposure to limited water supply, these varieties show increased alternative splicing events, higher expression of transcriptional factors, and the enrichment of genes and proteins associated with transcription, translation, protein synthesis, and ROS detoxification processes. Such molecular changes possibly contribute towards the drought resilience of sorghum plants by increasing the pool of regulatory and stress-responsive genes/proteins, increasing the functional diversity of resultant protein isoforms, and generating effective protective mechanisms against oxidative damage of cell constituents.

In contrast, drought-sensitive sorghum reduces metabolic activity, and increases protein degradation under water stress possibly as energy saving mechanisms in support of cell processes that are critical for plant survival. Epicuticular wax biosynthetic genes were also highly expressed under water deficit, emphasizing the importance of wax deposition in retarding cuticular water loss, and its reciprocal effect on water conservation. In addition, sorghum root physiology and metabolism under drought has emerged as a dynamic research area that deserves further study.

Indeed, the sorghum transcriptomics and proteomics studies reviewed in the current paper have employed a broad spectrum of experimental systems, analytical methods, sorghum varieties, and water stress treatments. Collectively, however, trends in constitutive, and drought-induced expression of genes/proteins involved in signaling, transcription, protein synthesis, secondary metabolism, protein stability, cellular transport and ROS detoxification processes have emerged as potential targets for future functional validation studies.

Because drought stress affects almost every metabolic pathway and process, a difficulty arises in interpreting the datasets that have been produced from these experiments. In particular, how does one identify key genes/proteins that are central to the adaptive response from peripheral changes that are a consequence of the reduced water availability? Even the use of near-isogenic sorghum lines in such experiments may not easily resolve this impediment. Refining the approach to include kinetic experiments focusing on early events after onset of drought is a potential avenue to identifying the critical components in sorghum adaptation to drought. This could be used in combination with forward genetic screens (employing mutagenized seeds) to identify components that are constitutively expressed and not responsive to drought stress. These challenges need to be resolved prior to incorporation of selected targets in breeding programs to produce more drought-resilient crops.

**Figure 3:**
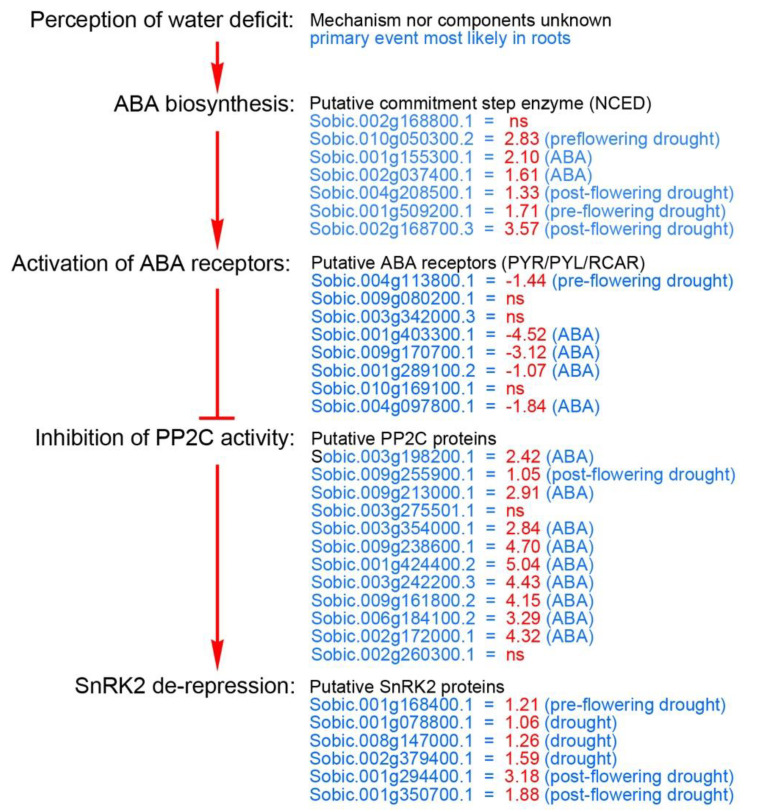
Transcriptomic responses of the putative sorghum ABA signaling module. Arabidopsis proteins were used with BLAST to search the Phytozome database for sorghum orthologs of (i) the commitment enzyme in the ABA biosynthesis pathway, (ii) the ABA receptor proteins—pyrabactin resistance (PYR)/PYR1-like (PYL)/regulatory component of ABA receptor (RCAR), (iii) the protein phosphatase 2C (PP2C) proteins, and (iv) the sucrose non-fermenting-1 (SNF1)-related protein kinase 2 (SnRK2). The response of each gene to drought or ABA was searched in the Genevestigator database [124] and the log2 ratio of treatment/control is provided in red font. The gene response data captured is from two field drought studies [19,74] and one laboratory-based ABA study [88]. Activation is denoted by a line with arrowhead, while protein inhibition is denoted by line ending with a T-junction. NCED, 9-*cis*-epoxycarotenoid dioxygenase; ns, no significant response.

In the meantime, vital clues of the sorghum response to drought stress can be assembled from comparison with other model species. A great deal of protein functional validation has been achieved using Arabidopsis loss-of-function mutants and transgenic overexpression plants. Of particular note is the drought-induced biosynthesis of ABA and downstream signaling. Perception of water deficits activates biosynthesis of ABA, which binds to its receptor complex PYR/PYL/RCAR (Pyrabactin Resistance 1/PYR1-Like/Regulatory Component of ABA Receptors) [122,123] to inhibit protein phosphatase 2C (PP2C) activity. PP2C inhibition triggers autophosphorylation of subclass III sucrose non-fermenting-1 (SNF1)-related protein kinase 2 (SnRK2) proteins, which then phosphorylate numerous downstream target genes important in adaptation to drought. Sorghum orthologs of Arabidopsis genes can be identified via homology searches and a putative sorghum ABA signaling module constructed by incorporating gene expression profiling data from Genevestigator (https://genevestigator.com/, accessed on 8 July 2021) [124] or similar databases. Figure 3 presents an example of such an exercise showing the response of sorghum orthologs of the ABA signaling genes, together with the enzyme catalyzing the commitment step in the biosynthesis of ABA. The transcriptomic results show activation of ABA biosynthesis, suppression of ABA receptor protein genes, and activation of PP2C and SnRK2 kinase genes. Suppression of ABA receptor genes possibly indicates transcriptional signal termination after PP2C inhibition has been achieved, while up-regulation of PP2C transcription could be a mechanism to re-establish normal homeostasis after SnRK2 activation has been completed. Analyses such as this one could provide useful hypotheses that can be tested using specific target genes for validation. A similar approach can be used to target different aspects of sorghum drought stress adaptation, such as biosynthesis of osmoprotectants.

## Figures and Tables

**Table 1:** Databases and computational resources/tools supporting sorghum “omics” research.

Database	Website *	Features	References
**Genomic resources/tools**			
NCBI	http://ncbi.nlm.nih.gov	Plant genomics resource	[25]
Phytozome	http://phytozome.jgi.doe.gov	Plant genomics resource	[24]
Gramene	https://www.gramene.org	Grass genomics resource	[31]
GreenPhylDB	http://www.greenphyl.org	Comparative and functional genomics in plants	[32]
PGSB PlantsDB	http://pgsb.helmholtz-muenchen.de/plant/plantsdb.jsp	Comparative genomics in plants	[33]
PlantGDB	http://www.plantgdb.org	Tools and resources for plant genomics	[34]
SorghumFDB	http://structuralbiology.cau.edu.cn/sorghum/index.html	Sorghum functional genomics with network analysis	[35]
SorGSD	http://sorgsd.big.ac.cn	Sorghum SNP data	[36]
DNApod	http://tga.nig.ac.jp/dnapod	Genome-wide DNA polymorphism datasets of plants	[37]
**Transcriptomic resources**			
Grassius	https://grassius.org/links.php	Grass transcription factors and gene promoters	[38]
GreenCircRNA	http://greencirc.cn	Plant circular RNAs (circRNAs)	[39]
KAAS	www.genome.jp/tools/kaas/	Pathway enrichment analyses of transcripts to classify spatial and temporal pathways	[40]
miRbase	http://www.mirbase.org	Plant microRNA (miRNA) data	[41]
Morokoshi transcriptome database	http://matsui-lab.riken.jp/morokoshi/Home.html	Sorghum transcriptome data	[42]
PlantTFDB	http://planttfdb.gao-lab.org/index.php	Plant transcription factors	[43]
PSRN	http://syslab5.nchu.edu.tw	Plant stress-specific transcriptome data	[44]
psRNATarget	http://plantgrn.noble.org/psRNATarget/	Potential miRNA sorghum target predictions	[45]
PtRFdb	http://www.nipgr.ac.in	Plant transfer RNA-derived fragments (tRFs) data	[46]
psRobot; Plant small RNA analysis toolbox	http://omicslab.genetics.ac.cn/psRobot/	Potential miRNA sorghum target predictions	[47]
UEA sRNA workbench	http://srna-workbench.cmp.uea.ac.uk	Various smallRNA (sRNA) tools	[48]
**Proteomic resources**			
CropPal	https://crop-pal.org	Protein subcellular location	[49]
ProtAnnDB	http://www.polebio.lrsv.ups-tlse.fr/ProtAnnDB/	Protein annotation	[50]
ExPASy	https://www.expasy.org/	Bioinformatics resources for proteomics	[51]
Uniprot	https://www.uniprot.org	Protein sequence and functional information	[52]
**Gene ontology/metabolic pathways**			
AgriGo	http://bioinfo.cau.edu.cn/agriGO/	Gene ontology of plant and agricultural species	[53]
KEGG	https://www.genome.jp/kegg/	Gene functional information	[54]
SorghumCyc	http://pathway.gramene.org/gramene/sorghumcyc.shtml	Metabolic pathways in sorghum	[31]

* Accessed on or before 18 July 2021.

**Table 2:** Summary of transcriptome studies of sorghum whole-plant systems under drought stress.

*S. bicolor* Variety with Known Drought Phenotype ^1^	Plant Tissue Sampled	Drought Experiment ^2^	Techniques Used	Summary of Key Findings ^3^	References
TX 430	Shoots	Slow dehydration stress of intact seedlings over 3M NaCl for 22 h.	Northern blotting, hybridization against a maize dehydrin probe.	Up-regulation of the dehydrin mRNA with increase in stress duration.	[85]
P954035—tolerant P721N—susceptible	Leaves Roots	Withholding water from seedlings and/or mature plants.	Northern blotting, hybridization against a maize dehydrin probe.	Up-regulation of the dehydrin mRNA in both seedlings and mature plants.	[86]
BTx623	Shoots Roots	20% PEG-8000 in a hydroponics set-up for 3 and 27 h. Other stresses: 125 μM ABA and 150 mM NaCl.	Microarray, qRT-PCR	ABA and PEG-induced DEGs greatly overlapped in shoots than roots. >100-fold increase in some growth—related genes (e.g., a sorghum acting depolymerization factor homolog, a beta-expansin gene). Up-regulation of genes involved in lipid metabolism, proline biosynthesis, protection (dehydrins/LEA proteins), ROS detoxification, post-translational modification, protein folding and turnover, transcription factors. Photosynthesis related genes were down-regulated.	[87]
BTx623	Shoots Roots	20% PEG-8000 in a hydroponics set-up for 27 h Another stress: 20 μM ABA.	RNA-seq, qRT-PCR	ABA treated samples showed a greater number of DEGs (both up-and down-regulated) than the PEG treatment. 12–30% of DEGs were common between PEG and ABA treatments, and tissue type. Genes for water stress-inducible protein 18 (WSI18), and LEAs, and dehydrins among the top five up-regulated DEGs in response to both treatments in roots and shoots, respectively. Up-regulation of DEGs involved in the following pathways, β-alanine betaine biosynthesis, amino acid metabolism, hormone biosynthesis and catabolism, plant defense (13-lipooxygenase and 13-hydroperoxide lyase), root disease response, abiotic stresses, cell growth processes, and regulation of transcriptional activity.	[88]
R16	Leaves	Withholding water from seedlings. Other stresses included heat shock and a combination of drought and heat.	Microarray, qRT-PCR	Highly up-regulated DEGs included those of LEA proteins, a proline biosynthetic enzyme *P5CS2*, a sodium ion transmembrane transporter *HKT1.* Genes involved in stress, response to water deprivation, response to ABA, amino acid regulation, fluid transport, regulation of photosynthesis were highly enriched. 380 genes were exclusively up-regulated in response to drought stress, including those associated with lipid transport, cell growth (expansins) and LEA proteins. Wax biosynthetic genes were up-regulated.	[73]
IS22330—tolerant IS20351—susceptible	Leaf meristem	Withholding water from seedlings.	RNA-seq, qRT-PCR	Higher constitutive expression of genes involved in the secondary metabolic process and GST activity in the drought-tolerant variety. Alternative splicing events increased in the drought-tolerant variety following stress. 1599 and 636 DEGs identified in drought-susceptible and drought-tolerant varieties, respectively. 559 and 78 DEGs were both unique to, and up-regulated in the drought-susceptible and drought-tolerant varieties, respectively. The susceptible variety metabolized carbohydrates while the tolerant one activated amino acid biosynthesis in response to drought.	[74]
South African landrace-LR6	Leaves	Progressive water stress and re-watering as follows: Mild stress (4 days of withholding water), Severe stress (6 days of withholding water), Re-watering for 5 h after 7 days of water stress.	Microarray, qRT-PCR	The number of DEGs in general, and that of transcription factors (TFs) were greatest under severe stress > mild stress > recovery conditions. TF-related genes were highly responsive to water deprivation and re-watering. Other examples of highly up-regulated DEGs include those for mitochondrial transcription termination Factor (mTERF), anion-transporting ATPase family and LEA proteins (mild stress); putative homology to Abscisic acid-Insensitive 2 (*ABI2*), mannosyltransferase, acid phosphatase/oxidoreductase/transition metal ion binding (severe stress); protein kinase, zinc ion binding, and chloroplast chaperonin 10 proteins (re-watered samples).	[89]
XGL-1—tolerant	Leaves, Roots	Withholding water from seedlings for 7 days, and sampling plant tissue from: mild drought (RWC ~ 60%), severe drought (RWC ~ 30%), re-watered (severe drought treatment plus re-watering for 2 days).	RNA-seq, qRT-PCR	510, 559 and 3,687 DEGs in leaf samples, and 3,368, 5,093, and 4,635 in root samples of mild drought, severe drought and re-watered plants. More DEGs in roots than leaves Most enriched GO terms of DEGs in both tissues included a response to stimulus, temperature stimulus, light intensity, ABA stimulus, and response to water deprivation. 20 and 130 DEGs common to all three treatments were involved in hormone stimulus pathway in leaves and roots, respectively. ABA biosynthetic genes were up-regulated in roots in response to drought but down-regulated in re-watered samples, while auxin signaling-related genes showed a reciprocal expression pattern. 4 and 44 TF genes responded to all three treatments in leaves and roots, respectively. Expansins were up-regulated during recovery from stress, but down-regulated during water deprivation.	[90]
BTx623 and SC56—resistant Tx7000 and PI-482662—sensitive	Whole seedlings	20% PEG-8000 applied on 8-day old seedlings growing in nutrient medium for 1 and 6 h.	RNA-seq, qRT-PCR	The total number of DEGs was greater at 6 h than 1 h. 42 and 129 DEGs were common to all varieties at 1 h and 6 h of stress, most of which were up-regulated. Early responses to PEG treatment included genes for hormone signaling and TFs. Late responses to PEG treatment included genes involved in secondary metabolism, heat shock and ROS detoxification processes. Examples of highly up-regulated genes common to all varieties at both time points include those of WSI18, alpha-amylase and GST. Examples of genes up-regulated only in the drought-resistant varieties include LEAs, TFs, signaling, and lipid metabolism-related genes.	[91]
SC56—tolerant Tx7000—susceptible	Leaves	Withholding water at anthesis for 13 days.	RNA-seq	Higher constitutive expression of genes in the tolerant variety than the susceptible variety, with enriched GO terms including translation, amino acid metabolism, carbohydrate metabolism, and cell homeostasis-related processes. 363 and 263 DEGs genes in the tolerant and susceptible variety, respectively. The tolerant variety responded to drought by up-regulating genes involved in translation, gene expression, metabolism, redox homeostasis, and drought regulatory genes, among others.	[92]
RTx430 BTx642	Leaves Roots	Plants grew over 17 weeks from seedlings to maturity and were sampled at weekly intervals. Watering withheld during pre-flowering and post-flowering growth stages. Other plants were re-watered after pre-flowering drought stress.	RNA-seq	A large-scale study with 198 leaf and 198 root transcriptomes. 10 272 DEGs observed accounting for 44% of all expressed genes. 10% of all expressed genes were modulated within the first week of drought stress treatments. Roots exhibited a greater number of DEG than leaves. Genotype specific differences were observed for both constitutive and drought-induced response. Tissue and developmental stage-specific differences in transcripts were observed. GST and proline biosynthetic genes were among the DEG with genotypic differences in expression.	[19]
M35-1—tolerant C43—susceptible	Leaves	Grown for 30 days after sowing. Water withheld until leaf relative water content of 60–65%.	TruSeq small RNA library prep and Illumina sequencing	96 miRNAs regulated specifically by drought stress: 32 up-, 49 down-, 15 genotype-contrasting regulation. The work demonstrated a genotype-dependent drought stress response, with the sensitive genotype having 17 drought differentially expressed miRNAs, with 18 in the tolerant line. tasi-RNA targetsmiR390-directed *TAS3* homologs and auxin response factors.	[93]
HSD 2945 HSD 3220 HSD 3221 HSD 3222 HSD 3223 HSD 3226 HSD 5299 HSD 5373 Arfa Gadamak—tolerant N98 Atlas	Leaves	Control plants watered every 10 days. Drought-stressed plants watered on 21-day interval.	qRT-PCR targeting 8 microRNA	Expression profiling of 8 microRNAs known to be down-regulated during abiotic stress (drought and control) across 11 sorghum genotypes.	[94]

^1^ The drought phenotypes of some sorghum genotypes are not stipulated in the reference citations. ^2^ Where multiple types of stresses were applied in a study, only results from the drought experiment are summarized in this table. ^3^ Due to the wide variations in experimental designs, and data analyses of the reviewed studies, it is beyond the scope of the current review paper to exhaustively list all key findings. For a comprehensive list of the results, readers are referred to the original research papers.

**Table 3:** Summary of proteomic studies of sorghum whole-plant systems under drought stress.

*S. bicolor* Variety with Known Drought Phenotype ^1^	Plant Tissues	Drought Experiment	Techniques Used	Summary of Key Findings ^2^	References
11434—tolerant 11431—susceptible	Leaves	Withholding water from seedlings until soil water potential of 1 MPa, Re-watering for 24 h.	2D-DIGE, MALDI-TOF-MS	Transcription, protein synthesis, protein destination and storage—related proteins were generally more up-regulated in the drought-tolerant varieties than the sensitive type in response to drought and/or re-watering. Proteases were up-regulated in the drought-sensitive variety in response to water deprivation.	[115]
SA1441—tolerant ICSB338—susceptible	Roots	Withholding water from seedlings for 8 days.	iTRAQ, qRT-PCR	Common and unique drought-responsive proteins were identified in the two varieties. The tolerant SA1441 up-regulated transcription, protein synthesis, protease inhibitors, signaling transduction, and transporter-related proteins in response to water deprivation. The sensitive ICSB338 down-regulated metabolism and protein synthesis but increased the proteolysis.	[71]
BTx623	Roots	20% PEG-6000 applied on 16-day-old seedlings growing on nutrient medium over 24 h.	2D-PAGE, CBB-G250 staining, MALDI-TOF-TOF MS	65 drought-responsive root proteins (up- and down-regulation) with a 2-fold change in abundance detected on gels. 52 of the 65 proteins were positively identified. The 3-topmost represented functional groups were energy and carbohydrate metabolism, antioxidant/defense and protein synthesis/processing/degradation. Up-regulated proteins were mainly involved in carbohydrate/energy/lipid metabolism, antioxidant functions, stress response (LEA like-proteins), protein synthesis and transport, regulation of transcription, and signaling functions.	[116]
EI9-tolerant Tabat-sensitive	Leaves	Withholding water from 14-day old seedlings for 7 days.	Nanoflow UPLC, MS	36 proteins were detected. Of these, 23 were drought-induced in either one or both sorghum varieties. Identified proteins were involved in a range of functions, including response to stress, metabolic processes, photosynthesis, cell wall biosynthesis/degradation, and fatty acid biosynthesis.	[117]

^1^ The drought phenotypes of some sorghum genotypes are not stipulated in the reference citations. ^2^ Due to the wide variations in experimental designs, and data analyses of the reviewed studies, it is beyond the scope of the current review paper to exhaustively list all key findings. For a comprehensive list of the results, readers are referred to the original research papers.
